# Systemic cytokine profiling reveals distinct pro-inflammatory signatures of arboviruses infections

**DOI:** 10.1007/s00705-026-06788-x

**Published:** 2026-11-14

**Authors:** Isabela Valim Sarmento, Carlos Henrique Fantecelle, Julia Sthefany Nunes Zordan, Bruna Caetano Pimenta, Suwellen Sardinha Dias de Azevedo, Isabela Ribeiro Rodrigues, Anna Clara Gregório Có, Jaqueline Pegoretti Goulart, Eric Arrivabene Tavares, Edson Oliveira Delatorre, Rodrigo Ribeiro Rodrigues, Luciana Polaco Covre, Daniel Cláudio Oliveira Gomes

**Affiliations:** 1 Núcleo de Doenças Infecciosas, Universidade Federal do Espírito Santo, Vitória, Brazil; 2 Núcleo de Biotecnologia, Universidade Federal do Espírito Santo, Vitória, Brazil; 3 Instituto Nacional de Ciência e Tecnologia em Mucosas e Pele, Belo Horizonte, MG Brazil; 4 Laboratório de Genômica e Ecologia Viral, Departamento de Patologia, Centro de Ciências da Saúde, Universidade Federal do Espírito Santo, Vitória, Espírito Santo Brazil; 5 Laboratório Central de Saúde Pública do Estado do Espírito Santo, Vitória, Brazil; 6 Escola Superior de Ciências da Santa Casa de Misericórdia de Vitória, Vitória, Espírito Santo Brazil; 7 Núcleo de Doenças Infecciosas/Núcleo de Biotecnologia, Universidade Federal do Espírito Santo – UFES, Av. Marechal Campos, 1468 - Maruípe, Vitória, Cep 29043-900 Brazil

## Abstract

Arboviruses are a growing public health concern in tropical regions, where dengue virus (DENV), chikungunya virus (CHIKV) and oropouche virus (OROV) frequently co-circulate and cause overlapping clinical manifestations. In this study, we characterised the systemic inflammatory response by quantifying 12 cytokines and chemokines using a multiplex immunoassay in 163 patients with laboratory-confirmed acute arboviral infection: DENV1 (*n* = 28) and DENV2 (*n* = 39) patients without warning signs, CHIKV (*n* = 42) and OROV (*n* = 54). Thirty-one healthy controls (HC, *n* = 31) were also included. Clinically, arthralgia was the only feature that reliably distinguished the infections, occurring in 57.1% of CHIKV patients compared with 7.4–30.8% in the other groups, whereas fever, headache and myalgia overlapped substantially across all four. Immunologically, OROV induced a selective response with elevated IL-18 and CCL2/MCP-1 alongside reduced IL-1β, TNF-α, IL-12p70, IL-23 and IL-17 A; DENV1 and DENV2 elicited broad activation of pro-inflammatory and antiviral mediators, with DENV1 engaging a wider range of cytokines than DENV2; and CHIKV elevated IFN-α2, IFN-γ, IL-18, IL-6, IL-8, MCP-1 and IL-17 A. Cytokine network analysis revealed virus-specific patterns of immune connectivity, with OROV showing the most coordinated cytokines and chemokine response and CHIKV the most fragmented. Circulating cytokine concentrations were largely independent of viral load across all viruses, indicating that systemic inflammation during acute arboviral infection is driven primarily by host immune activation rather than by viral replication kinetics. These findings establish distinct immunological signatures for DENV, CHIKV, and OROV that may support differential diagnosis and inform the development of pathogen-specific therapeutic strategies.

**Supplementary Information:**

The online version contains supplementary material available at https://doi.org/10.1007/s00705-026-06788-x.

## Introduction

Arthropod-borne viruses (arboviruses) represent a significant and growing global health burden, with disproportionate impact in tropical and subtropical regions [1]. Transmitted by hematophagous arthropods, these viruses span diverse taxonomic families, including *Flaviviridae*, *Togaviridae*, and *Peribunyaviridae*, and are responsible for a broad spectrum of febrile and potentially life-threatening illnesses in humans [2–4]. Brazil has emerged as a major epicenter of arboviral disease, where dengue virus (DENV), chikungunya virus (CHIKV), and oropouche virus (OROV) constitute clinically and epidemiologically relevant pathogens [5].

Dengue remains the most prevalent arboviral disease worldwide, with an estimated 390 million infections occurring annually and approximately 20,000 deaths reported each year [6, 7]. Chikungunya virus, following its re-emergence in 2006, has continued to impose a substantial public health burden in endemic regions, with an estimated 1.39 million suspected cases reported to date, reflecting its sustained transmission dynamics and long-term impact on affected populations [8]. More recently, oropouche virus has garnered increasing attention as an emerging threat, with more than 11,600 confirmed cases recorded across the Americas by late 2024 [9, 10]. Critically, documented cases of vertical transmission leading to congenital anomalies and fetal death, as well as fatal outcomes in adults, have underscored the severity and underrecognized potential of this historically neglected arbovirus [11, 12].

Although most arboviruses fever share overlapping epidemiological features and often present with clinically indistinguishable acute manifestations, including fever, myalgia, arthralgia, and rash, their underlying pathogenic mechanisms and long-term clinical outcomes differ substantially [13]. Increasing evidence indicates that the host immune response plays a central, and at times decisive, role in determining disease severity, beyond what can be attributed to viral factors alone. In particular, dysregulated production of inflammatory cytokines and chemokines has emerged as a key pathogenic mechanism in severe arboviral disease, contributing to vascular dysfunction, tissue injury, and adverse clinical outcomes in both dengue and chikungunya infections [14].

Despite important advances in understanding the immunopathogenesis of individual arboviral infections, comparative analyses of the immune and inflammatory responses elicited by DENV, CHIKV, and OROV, three co-circulating arboviruses of major public health relevance in Brazil remain poorly understood.

In this exploratory study, we characterized the systemic cytokine and chemokine profiles during the acute phase of dengue, oropouche, and chikungunya virus infections. Our primary objective was to identify inflammatory mediators altered in patients relative to healthy controls and to establish virus-specific inflammatory signatures capable of distinguishing these arboviral infections. As a secondary objective, we investigated the correlation between cytokine levels and viral load to determine whether the magnitude of the systemic immune response reflects viral replication kinetics during early infection. Our findings show pronounced alterations in pro-inflammatory cytokines and chemokines across all three infections. Network analysis demonstrated marked differences in cytokine connectivity among the infections, indicating distinct patterns of immune activation despite their overlapping clinical presentations. Circulating cytokine concentrations were largely independent of viral load across all arboviruses, which suggests that the systemic inflammatory response during the acute phase is driven mainly by host immune activation rather than by the extent of viral replication.

Taken together, these findings contribute to a more comprehensive understanding of the distinct immunological and inflammatory signatures induced by DENV, CHIKV, and OROV, which may contribute to differential diagnosis and to the rational design of pathogen-specific therapeutic strategies.

## Materials and methods

Serum samples were obtained from peripheral blood collected from 31 healthy controls, 54 OROV patients, 42 CHIKV patients, 28 DENV1 patients, and 39 DENV2 patients. Dengue patients included those without warning signs and those who did not progress to severe dengue [15]. Patients were recruited from primary healthcare services across the state of Espírito Santo, Brazil. Diagnosis was established at the Central Laboratory of Espírito Santo (LACEN-ES/SESA) based on clinical and laboratory confirmation, with all patients testing positive for only the respective arboviral disease, that is DENV1, DENV2, CHIKV or OROV, by RT-qPCR. In addition, all participants tested negative for zika virus (ZIKV), and the remaining arboviruses in this study, using commercial ZDC RT-qPCR kits, and mayaro virus (MAYV) by RT-PCR [16]. The control group (*n* = 31) consisted of healthy, age- and sex-matched individuals (HC), comprising 70% males and 30% females, closely mirroring the distribution observed in the patient cohorts (73% male, 27% female). All controls resided in the same endemic area were clinically asymptomatic for any arbovirus-like illness, and tested negative for oropouche, dengue and chikungunya virus infections in the moment of the study. The study was approved by the Institutional Human Research Ethics Committee (protocol no. 84698324.7.0000.5064).

### Cytokine and chemokine assessment

Serum levels of chemokines (IL-8/CXCL8 and MCP-1/CCL2) and cytokines (IL-1β, IFN-α2, IFN-γ, TNF-α, IL-6, IL-10, IL-12p70, IL-17 A, IL-18 and IL-23) from health control (HC), DENV1-, DENV2-, CHIKV- and OROV-positive patients enrolled in this study were quantified using the LEGENDPlex™ Human Inflammation Panel 1 (BioLegend, cat# 740808). Samples were processed according to the manufacturer’s instructions and immediately acquired on a CytoFLEX LX flow cytometer (Beckman Coulter). Data were analyzed using the LEGENDPlex™ Data Analysis Software Suite, and results are expressed in pg/mL.

### Statistics

R (version 4.4.3) was used to perform all statistical analyses. Outliers were identified and excluded using an unscaled robust Median Absolute Deviation (MAD) score:

$$\:{MAD\:Score}_{i}\:=\:\frac{{x}_{i}\:-\:median\left(x\right)}{median\left(|{x}_{i}-\:median\left(x\right)|\right)}$$

where values with absolute MAD score higher than 3 were excluded from subsequent analyses, as available in the Supplementary Table 1. Normality of the data distribution was evaluated using the Shapiro–Wilk test, where we found all data to be non-normally distributed. To compare between HC, OROV, CHIKV, DENV1 and DENV2 infected groups, the Wilcoxon and Pairwise Wilcoxon Rank-sum tests for non-parametric and unpaired data were used and p-values were adjusted for multiple testing using the Benjamini-Hochberg method (BH) considering all tests performed. Effect sizes were estimated using the Hodges-Lehmann Median Difference (Supplementary Table 1) method:

$$\:\stackrel{\sim}{\theta\:}=median\:(HC-OROV)$$

with 95% confidence intervals (CI). For correlation network analyses between all cytokines, the Spearman’s Rank Correlation Coefficient was used. In the HC group, multiple comparisons were also corrected using the BH procedure. To determine the influence of the viral infections in the relationships observed in healthy individuals, we calculated a difference network by subtracting the HC correlation matrices from the corresponding group of interest:

$$\:\varDelta\:\rho\:={\rho\:}_{i}-{\rho\:}_{HC}$$

To determine the statistical significance of the observed differences, a nonparametric, independent samples, bootstrapping procedure with 1,000 permutations and p-values were adjusted using the BH method and the 95% CIs were obtained from the bootstrap percentiles. The correlation between cytokine concentrations and -CT values were calculated using a similar bootstrapping procedure, but a partial Spearman’s correlation controlling for days after symptoms. This time, 95% CIs were obtained using the bias-corrected and accelerated (BCa) method in the *boots* R package. All plots were generated using *ggplot2* and *ggraph*. Statistical significances are represented by corrected p-values coded using the following convention throughout the manuscript: *p* < 0.05 (*), *p* < 0.01 (**), *p* < 0.001 (***), and *p* < 0.0001 (****).

## Results

### Patients profile and clinical presentation in arboviral infection

The study cohort comprised 163 patients with laboratory-confirmed acute arboviral infection, distributed across four infection groups: chikungunya virus (CHIKV, *n* = 42), oropouche virus (OROV, *n* = 54) and dengue virus serotype 1 (DENV1, *n* = 28), Dengue virus serotype 2 (DENV2, *n* = 39). All dengue patients were classified as having dengue without warning signs and did not progress to severe dengue [15]. The mean age of patients was comparable across groups, with 48.3 ± 19.6 in CHIKV infected patients, 47.4 ± 17.3 in DENV1, 38.4 ± 18.7 in DENV2 and 44.2 ± 16.6 in OROV infected patients. DENV2 presented the lowest mean age, although the age range of the group, 14 to 85 years, was similar across all groups (Table 1). The duration of symptoms prior to diagnosis ranged from 1 to 4 days across all infection groups, except CHIKV in which patients of 1 to 6 days after symptom onset were included.

**Table 1:** Clinical characteristics of chikungunya, oropouche and dengue 1 and dengue 2 infected patients

Group		Gender				
	*n*	Female (%)	Male (%)	Days after symptoms onset	Age (mean ± SD [min-max])	Symptoms (% [*n*])
CHIKV	42	64.3%	35.7%	1–6	48.3 ± 19.5 (6–88)	85.7% Myalgia [36], 83.3% Fever [35], 71.4% Headache [30], 57.1% Intense arthralgia [24], 28.6% Back pain [12], 28.6% Retro-orbital pain [12], 26.2% Arthritis [11], 23.8% Nausea [10], 9.5% Exanthema [4], 7.1% Arterial hypertension [3], 7.1% Diabetes [3], 7.1% Vomiting [3], 4.8% Conjunctivitis [2], 2.4% Leukopenia [1], 2.4% Petechiae [1]
DENV1	28	57.1%	42.9%	1–4	47.4 ± 17.3 (12–86)	96.4% Headache [27], 92.9% Fever [26], 67.9% Myalgia [19], 42.9% Retro-orbital pain [12], 39.3% Nausea [11], 28.6% Intense arthralgia [8], 25.0% Back pain [7], 21.4% Arterial hypertension [6], 14.3% Arthritis [4], 14.3% Vomiting [4], 7.1% Diabetes [2], 3.6% Exanthema [1]
DENV2	39	61.5%	38.5%	1–4	38.4 ± 18.7 (14–85)	92.3% Fever [36], 82.1% Myalgia [32], 76.9% Headache [30], 41.0% Retro-orbital pain [16], 33.3% Back pain [13], 33.3% Nausea [13], 30.8% Intense arthralgia [12], 17.9% Vomiting [7], 15.4% Arthritis [6], 7.7% Arterial hypertension [3], 5.1% Exanthema [2], 5.1% Leukopenia [2], 2.6% Hospitalization [1], 2.6% Petechiae [1]
OROV	54	31.5%	68.5%	1–4	44.2 ± 16.6 (13–83)	90.7% Fever [49], 87.0% Headache [47], 74.1% Myalgia [40], 33.3% Back pain [18], 33.3% Retro-orbital pain [18], 33.3% Vomiting [18], 29.6% Nausea [16], 13.0% Arthritis [7], 7.4% Intense arthralgia [4], 5.6% Arterial hypertension [3], 5.6% Leukopenia [3], 3.7% Diabetes [2], 3.7% Tourniquet test [2], 1.9% Conjunctivitis [1]

The clinical presentation was characterized by a constellation of systemic symptoms with varying frequencies across infection groups. Fever was the most prevalent symptom overall, affecting 83.3% of CHIKV-, 92.9% of DENV1-, 92.3% of DENV2-, and 90.7% of OROV-infected patients. Headache was the second most frequently reported symptom, present in 71.4%, 96.4%, 76.9%, and 87.0% of CHIKV-, DENV1-, DENV2-, and OROV-infected patients, respectively. Myalgia was reported by 85.7% of CHIKV-, 67.9% of DENV1-, 82.1% of DENV2-, and 74.1% of OROV-infected patients. Back pain was similarly prevalent across groups, reported in 28.6%, 25.0%, 33.3%, and 33.3% of CHIKV-, DENV1-, DENV2-, and OROV-infected patients, respectively, suggesting that this symptom represents a non-discriminating feature of acute arboviral disease. Retro-orbital pain was reported in 28.6% of CHIKV-, 42.9% of DENV1-, 41.0% of DENV2-, and 33.3% of OROV-infected patients, with a somewhat higher prevalence in Dengue-infected individuals. Gastrointestinal symptoms were less prevalent than systemic manifestations but were present across all infection groups. Nausea was reported in 23.8%, 39.3%, 33.3%, and 29.6% of CHIKV-, DENV1-, DENV2-, and OROV-infected patients, respectively, while vomiting was infrequent across all groups, with frequencies of 7.1%, 14.3%, 17.9% in CHIKV-, DENV1- and DENV2 infected patients respectively but was slightly more frequently observed in OROV-infected patients, with 33.3% of patients exhibiting the symptom. Among the musculoskeletal manifestations, intense arthralgia represented the most clinically discriminating symptom across the infection groups. It was reported in 57.1% of CHIKV-infected patients, compared with 28.6%, 30.8%, and 7.4% in DENV1-, DENV2-, and OROV-infected patients, respectively.

### Systemic cytokine and chemokine profiles in patients with acute oropouche, dengue, and chikungunya virus infections

**Fig. 1:**
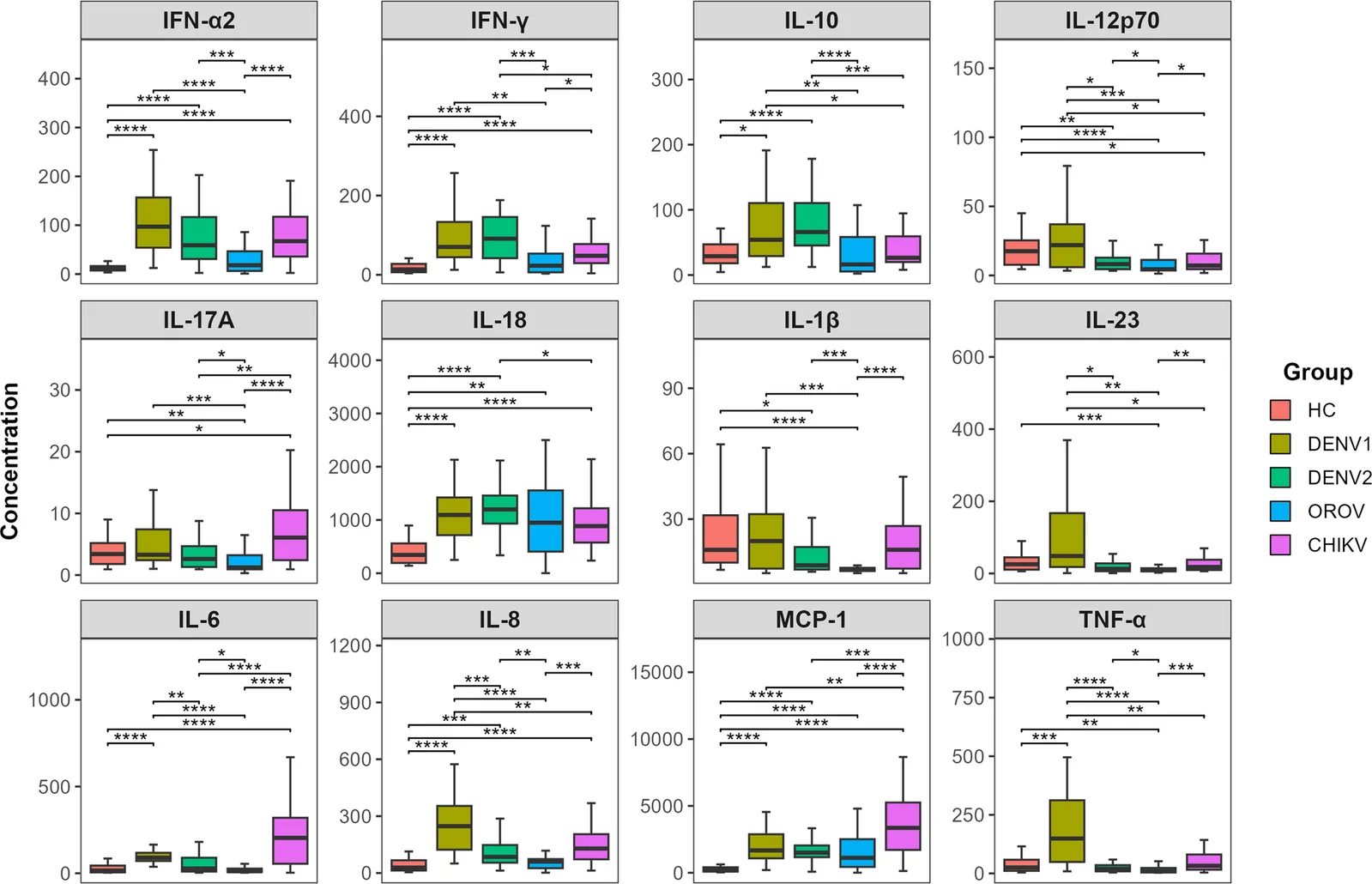
Cytokine and chemokine profile in Dengue, Oropouche and Chikungunya virus-infected patients. Boxplots showing serum cytokine and chemokine concentrations (pg/mL) in healthy controls (HC) and patients with dengue virus serotype 1 (DENV1), dengue virus serotype 2 (DENV2), oropouche virus (OROV), and chikungunya virus (CHIKV) infections. Statistical significance was assessed using the Wilcoxon rank-sum test, and *p*-values were adjusted using the Benjamini-Hochberg false discovery rate (FDR) procedure (*p* < 0.05, *p < 0.01, **p < 0.001, and *p < 0.0001)

The acute-phase systemic release of inflammatory cytokines and chemokines is a hallmark of arboviral pathophysiology [17, 18]. To characterise and compare these responses across four clinically relevant arboviruses in Brazil, we measured circulating mediator concentrations in patients with laboratory-confirmed acute infection by oropouche virus (OROV), dengue virus serotypes 1 and 2 (DENV1 and DENV2), and chikungunya virus (CHIKV), alongside healthy controls (HC). Quantification was performed using a multiplex immunoassay panel covering type I and type II interferons (IFN-α2 and IFN-γ), interleukins (IL-1β, IL-6, IL-8, IL-10, IL-12p70, IL-17 A, IL-18 and IL-23), the monocyte chemoattractant protein MCP-1/CCL2, and TNF-α (Fig. 1). OROV-infected patients showed a comparatively restrained inflammatory signature. Only IL-18 and MCP-1/CCL2 were significantly elevated relative to HC, while IL-1β, IL-12p70, IL-17 A, IL-23 and TNF-α were significantly reduced. The remaining mediators, including IL-6, IL-8, IL-10, IFN-α2 and IFN-γ, did not differ significantly from HC. DENV1 and DENV2 showed a broader inflammatory response than OROV, with both serotypes significantly elevated over HC for IFN-α2, IFN-γ, IL-10, IL-18, IL-8 and MCP-1. TNF-α, and IL-6 were significantly elevated in DENV1 but not in DENV2, indicating that although the two serotypes share a broadly similar profile, DENV1 engaged a somewhat wider range of mediators. CHIKV-infected patients showed the most distinct profile of the four groups. IFN-α2, IFN-γ, IL-18, IL-6, IL-8, MCP-1 and IL-17 A were all significantly elevated relative to HC, with IL-17 A and IL-6 reaching some of the highest concentrations observed across all groups, consistent with the association between Chikungunya and Th17-skewed inflammation reported previously [19]. IL-12p70, in contrast, was significantly reduced relative to HC, a pattern shared with all other groups except DENV1.

To characterise the kinetics of the inflammatory response in more detail, we performed a complementary temporal analysis of circulating mediator concentrations, stratifying patients according to the interval between symptom onset and diagnosis (1–2 days and 3–4 days for OROV, DENV1 and DENV2; 1–2, 3–4 and 5–6 days for CHIKV) (Supplementary Fig. 1). This analysis revealed distinct, pathogen-specific dynamics of mediator production over the course of the acute phase. In OROV-infected patients, the inflammatory signature was elevated for IL-18 and MCP-1/CCL2 alongside reduced TNF-α, that was established within the first 1–2 days of symptoms and remained essentially unchanged through days 3–4 (Supplementary Fig. 1A). This early signature also extended to IL-12p70, IL-1β and IL-23, all of which were significantly reduced relative to HC from the earliest timepoint onward, and IL-17 A which was reduced on the 1–2 days group but showed a reversal trend towards HC levels. IL-6, IL-8, IFN-α2, IFN-γ and IL-10 did not differ significantly from HC at either timepoint (Supplementary Fig. 1A). CHIKV-infected patients showed a broader and more dynamic pattern. IFN-α2, IFN-γ, IL-18, IL-6, IL-8 and MCP-1 were all significantly elevated relative to HC from 1 to 2 days after symptom onset and remained elevated through 3–4 days (Supplementary Fig. 1B). MCP-1 and IL-18 additionally differed significantly between the 1–2 days and 5–6 days groups, indicating a sustained increase across the acute phase rather than an early production only. IL-17 A followed a different pattern, with significant increases only at 1–2 days, with varying degrees of production in the remaining groups. IL-12p70 reached reduced significance only at 3–4 days. IL-10, IL-1β, IL-23 and TNF-α did not differ significantly from HC at any timepoint assessed (Supplementary Fig. 1B). In DENV-infected patients, both serotypes showed a broad multi-mediator response established early and largely sustained. IFN-α2, IFN-γ, IL-18 and MCP-1 were significantly elevated relative to HC at both 1–2 and 3–4 days in both DENV1 and DENV2. IL-6 and IL-8 followed this pattern in DENV1, whereas in DENV2 only IL-8 reached significance at both timepoints and IL-6 did not differ from HC at all (Supplementary Fig. 1C and D). TNF-α was significantly elevated at both timepoints in DENV1 but not in DENV2, and IL-12p70 reached significance only at 1–2 days in DENV2 and not at all in DENV1. IL-10 was significantly elevated in both serotypes, reaching significance earlier in DENV2 at 1–2 days (Supplementary Fig. 1C and D). Taken together, the two dengue serotypes show a broadly convergent early and sustained inflammatory profile, though they diverge in a subset of mediators, including TNF-α and IL-12p70.

### Interconnected cytokine networks and correlative patterns

**Fig. 2:**
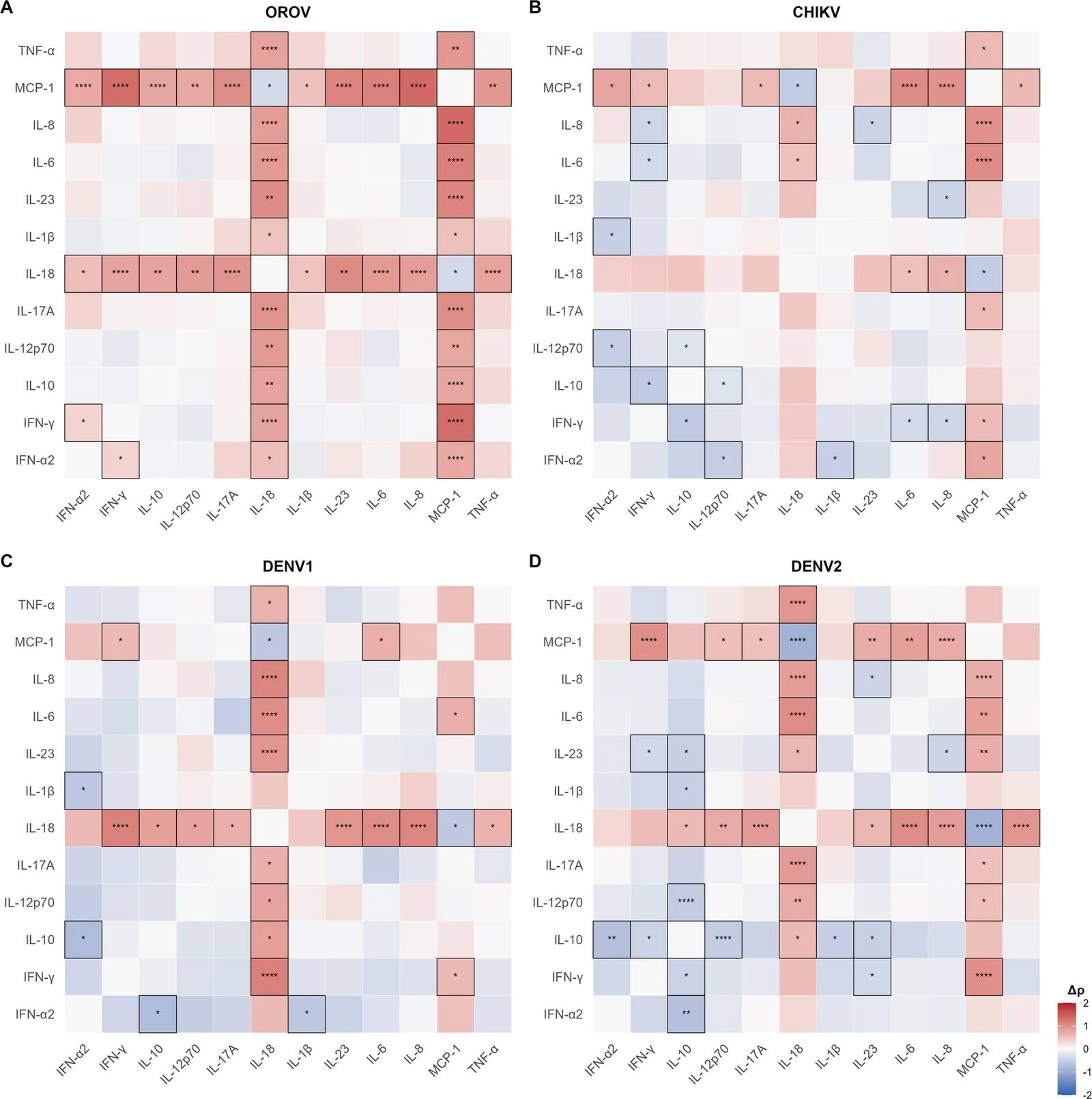
Cytokine correlation network analysis in Oropouche, Chikungunya and Dengue virus-infected patients. Difference network heatmaps showing the change in Spearman correlation coefficients among all measured cytokines and chemokines between oropouche (**A**), chikungunya (**B**), dengue 1 (**C**) and dengue 2-infected patients (**D**) versus controls ($\:\varDelta\:\rho\:={\rho\:}_{OROV}-{\rho\:}_{HC}$). The scale ranges from blue (negative difference in correlation, − 2.0) to red (positive difference in correlation, 2.0), with statistical significance measured by nonparametric bootstrapping with 1,000 permutations and Benjamini-Hochberg corrected denoted as *: *p* < 0.05, **: *p* < 0.01, ***: *p* < 0.001, ****: *p* < 0.0001

Network correlation analysis revealed distinct cytokine-chemokine rewiring patterns among the four arboviral infections, highlighting differences in their immune activation profiles (Fig. 2 and Supplementary Fig. 2B) when compared the correlations observed in HC (Supplementary Fig. 2A). OROV exhibited the most coordinated systemic response, characterized by a widespread shift in correlations among inflammatory mediators. CCL2 (MCP-1) (median Δρ = 0.924, highest p-value = 0.0343) and CXCL8 (IL-8) (median Δρ = 0.82, highest p-value = 0.0425) emerged as central hubs, showing extensive associations with multiple cytokines, including IFN-α2, IFN-γ, IL-1β, IL-6, IL-10, IL-12p70, IL-17 A, IL-18, IL-23 and TNF-α, in contrast with the correlations observed in healthy controls, where both CCL2 (median ρ = -0.47, median p-value = 0.00557) and CXCL8 (median ρ = -0.42, median p-value = 0.0101) were negatively correlated with the majority of other cytokines.

In contrast, Chikungunya displayed a more fragmented network, with fewer and weaker correlations involving MCP-1, IL-8, IL-1β, IL-18 and IL-23, alongside negative correlations involving IL-10. DENV1 showed an intermediate profile, with IL-18 acting as the principal correlation hub through positive associations with IL-1β, IL-23 and IFN-γ. In complement, --2 exhibited the most intense pro-inflammatory network, with IL-18 serving as the dominant central mediator through strong correlations with IL-1β, IL-23, IL-6, IL-8 and MCP-1. IL-10 correlated negatively with IFN-α2 in both DENV1 and DENV2, whereas the negative correlation with IFN-γ was restricted to DENV2.

### Cytokine response uncoupled from viral load

**Fig. 3:**
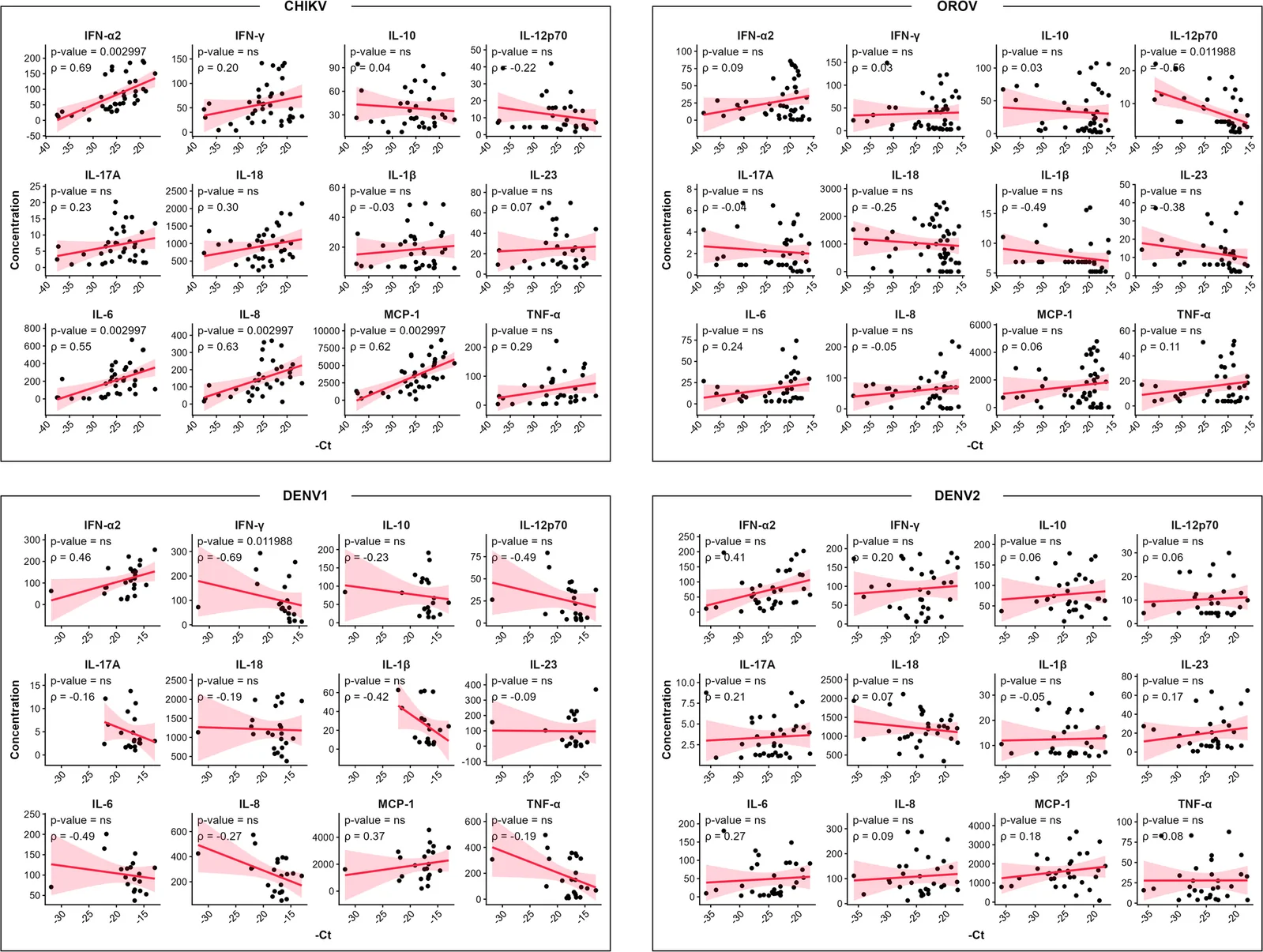
Correlation analysis between cytokine concentrations and viral load in Chikungunya, Oropouche and Dengue virus-infected patients. Scatter plots illustrating the relationship between serum cytokine/chemokine levels and viral load, as determined by RT-qPCR cycle threshold (Ct) values. Each dot represents an individual patient sample; red lines indicate linear regression with 95% confidence intervals (CIs) for the fitted regression line. Spearman’s rank correlation coefficients (ρ), and corresponding p-values (Benjamini-Hochberg adjusted) are shown in text for each cytokine and were calculated using non-parametric bootstrap resampling procedure with 1,000 permutations

In some arboviral infections, inflammatory intensity is generally correlated with viral load [20–22]. To assess whether this association holds across the four arboviruses studied here, we first assessed the difference between days after symptom onset and cycle threshold (Ct) values as an indirect measure of viral load. In this analysis, we only observed a difference in DENV2 group, where a reduction in viral load was seen after 2 days of symptoms (Supplementary Fig. 3). Since this difference was observed, we performed partial (controlling for days after symptoms) Spearman correlation analyses between Ct and the concentrations of circulating inflammatory mediators measured in patient samples (Fig. 3). Distinct virus-specific correlation patterns emerged. CHIKV showed the strongest association between viral load and systemic inflammation, with significant positive correlations between Ct and IFN-α2 (ρ = 0.69, *p* = 0.003), IL-6 (ρ = 0.55, *p* = 0.003), IL-8 (ρ = 0.63, *p* = 0.003) and MCP-1 (ρ = 0.62, *p* = 0.003), the remaining eight mediators showed no significant association. The other groups demonstrated an otherwise more restricted pattern. DENV1 showed a negative association between Ct and IFN-γ (ρ = -0.60, *p* = 0.012), with all other mediators showing weak, non-significant trends, while the OROV group presented significant negative correlations for IL-12p70 (ρ = -0.56, *p* = 0.012) and for IL-1β (ρ = -0.49, *p* = 0.024). DENV2 was the only group with no significant correlation for any of the twelve mediators assessed, all trends being weak and non-significant. Taken together, these results show that the relationship between viral load and systemic cytokine production is not uniform across arboviruses.

### Integrated overview of the virus-specific inflammatory signature

**Fig. 4:**
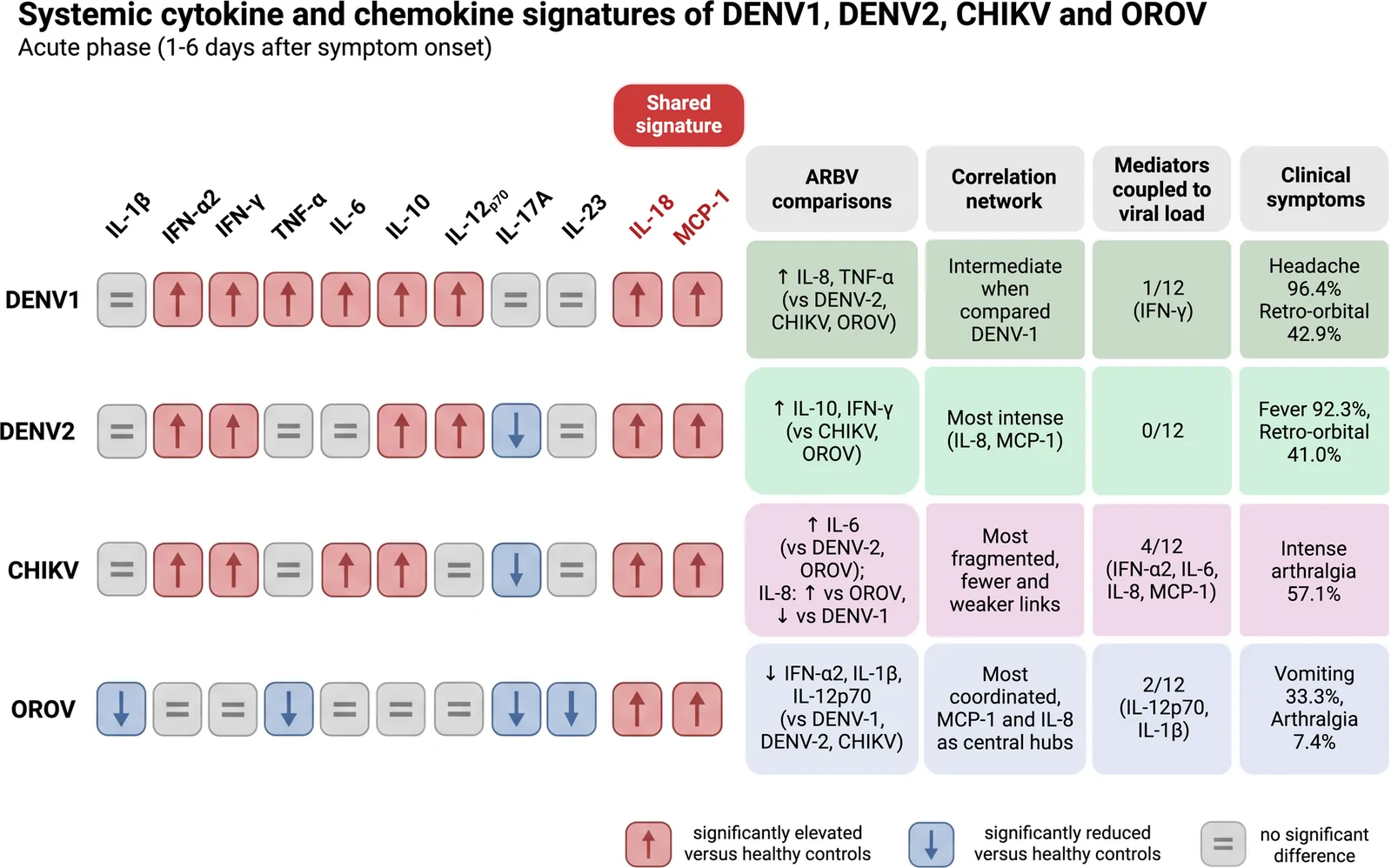
Summary of the systemic cytokine and chemokine signatures of DENV1, DENV2, CHIKV and OROV infection. Direction of change relative to healthy controls is shown for each of the 12 mediators measured during the acute phase (1 to 6 days after symptom onset): elevated (red), reduced (blue) and not significantly different (grey). The panels on the right summarise, for each virus, the topology of the correlation network (Fig. 2 and Supplementary Fig. 3B), the number of mediators showing a significant partial correlation with viral load after controlling for days after symptom onset (Fig. 3), and the most frequent or most discriminating clinical features (Table 1). Significance was determined using Wilcoxon rank-sum tests with Benjamini-Hochberg correction. Created in BioRender. Sarmento, I. (2026) https://BioRender.com/8bg31kq

Figure 4 summarises the findings described above. IL-18 and MCP-1/CCL2 formed a conserved core, being the only mediators elevated in all four infections. The remaining changes differed mainly in direction: OROV showed reductions in 5 of its 7 altered mediators, all 8 altered mediators were elevated in DENV1, and DENV2 and CHIKV each showed a single reduction, in IL-12p70.

The figure also highlights contrasts between infections. DENV1 and DENV2 diverged only in TNF-α and IL-6, and IL-17 A was elevated exclusively in CHIKV. Correlation network topology was most coordinated in OROV and most fragmented in CHIKV, and coupling to viral load was strongest in CHIKV and absent in DENV2.

## Discussion

Arboviral diseases are an increasing public health challenge in Brazil, where the co-circulation of Dengue, Chikungunya, Zika and Oropouche viruses complicates both clinical diagnosis and epidemiological surveillance. In this exploratory study, we investigated 163 patients with laboratory-confirmed acute arboviral infection, comprising CHIKV (*n* = 42), DENV1 (*n* = 28), DENV2 (*n* = 39), and OROV (*n* = 54). All dengue patients were classified as having dengue without warning signs and did not progress to severe dengue [15]. Participants were predominantly adults, with comparable mean ages across groups (38.4 ± 18.7 to 48.3 ± 19.5 years), and all samples were collected within 1–6 days of symptom onset, corresponding to the acute viraemic phase. Nonetheless, ages within groups were widely distributed from 6 to 88, 13 to 83, 12 to 86 and 14 to 85 in CHIKV, OROV, DENV1 and DENV2 groups respectively. This relatively continuous age distribution, in conjunction with the number of samples we evaluated, limits the extent to which we can assess how demographic factors could account for the differences described below. Since the wide age range and narrow sampling window preclude any assessment of age-dependent immune responses or of cytokine dynamics beyond the early stage of infection, these factors remain as important limitations of this study.

Among the clinical manifestations evaluated, intense arthralgia was the most discriminative feature, occurring in 57.1% of CHIKV-infected patients compared with 28.6% in DENV1, 30.8% in DENV2 and only 7.4% in OROV. This is consistent with the established tropism of CHIKV for joint tissue, including synovial fibroblasts and chondrocytes, which supports local viral replication and sustained inflammatory mediator production within the joint itself [23, 24]. In OROV, intense arthralgia remained infrequent despite a high prevalence of fever, headache and myalgia and despite clear evidence of coordinated systemic cytokine activation, indicating that systemic immune activation and joint-specific inflammation are uncoupled in this infection, unlike in CHIKV, where the two processes appear to occur together frequently. Fever, headache and myalgia were common across all four infections and therefore offered limited discriminatory value, whereas intense arthralgia seemed to separate CHIKV from the other three. These clinical differences parallel the distinct inflammatory profiles described below, but the overlap in the remaining acute manifestations means that clinical presentation alone cannot reliably differentiate between these infections, reinforcing the need for laboratory confirmation in routine diagnosis.

The cytokine profiling further demonstrated virus-specific patterns of immune activation. OROV induced a comparatively restrained and selective response, with IL-18 and CCL2/MCP-1 significantly elevated relative to controls, while TNF-α, IL-12p70, IL-17 A, IL-1β and IL-23 were significantly reduced. This combination of selective elevation and broad suppression of several mediators points to innate activation channelled preferentially through inflammasome-associated pathways rather than generalised inflammatory amplification: elevated IL-18 is compatible with inflammasome signalling, and elevated CCL2/MCP-1 suggests recruitment of circulating monocytes to infected tissue, as previously described [25]. DENV1 and DENV2, by contrast, elicited a broader response spanning innate and adaptive mediators, with concurrent elevation of IFN-α2, IL-18, IL-8, MCP-1/CCL2, IFN-γ and IL-12p70 in line with previous reports of robust antiviral and pro-inflammatory activation in acute dengue, a profile associated with endothelial activation, vascular dysfunction and disease severity, despite showing an increase in IL-10 production [26, 27]. CHIKV showed a pattern dominated by IL-6 and IL-17 A, consistent with previous evidence implicating Th17-associated immunity in chikungunya pathogenesis; both cytokines are established mediators of neutrophil recruitment, tissue inflammation and bone remodelling, and may contribute to the high frequency of arthralgia observed in this cohort. In addition, mediators such as IL-6, IL-10, IL-18 and MCP-1/CCL2, have been proposed as markers of dengue severity, and IFN-γ, IL-12p70 and IL-18 participate in the Th1 axis associated with host resistance and immunopathology [17, 25, 27]. Our data are consistent with those observations at the level of the acute inflammatory profile, but the cross-sectional design and the absence of outcome data preclude any assessment of their predictive performance. Establishing whether the virus-specific signatures described here precede clinical deterioration would require prospective cohorts with defined severity endpoints and serial sampling.

An additional point to consider is the presence of participants with reported comorbidities, including diabetes and arterial hypertension. As these conditions may influence systemic inflammatory profiles, they could represent potential confounding factors in comparative cytokine analyses. However, no statistically significant differences were detected for any of the measured mediators between participants with and without reported comorbidities within each infection group (data not shown), suggesting that these conditions did not substantially affect the comparisons observed here. Nonetheless, this analysis was limited by the small number of participants with comorbidities, and larger studies specifically designed to address this question are needed.

In our experiments, the three infections also differed in inflammatory kinetics. OROV established a stable cytokine signature within the first 1–2 days of symptoms that remained essentially unchanged through days 3–4, except for IL-17 A, whereas dengue sustained a broad multi-mediator response throughout the acute phase. CHIKV showed a more progressive course in which IL-17 A rose from a modest elevation to a significant increase, indicating ongoing amplification of Th17-mediated inflammation as infection advances. This progressive Th17 amplification may plausibly extend into the chronic phase, providing a pathogenic link between the early cytokine response and the persistent arthralgia that characterises long-term chikungunya sequelae, although this cohort’s cross-sectional design cannot establish that link directly. The sustained, multi-mediator profile seen in dengue, combining Th1 activation (IFN-γ, IL-12p70), broadly pro-inflammatory signalling (TNF-α, IL-6, IL-8, IFN-α2, IL-18) and monocyte recruitment (MCP-1/CCL2), is compatible with the inflammatory trajectory described in patients who progress to severe [28, 29]. Taken together, these kinetic findings indicate that OROV, CHIKV and DENV follow distinct inflammatory trajectories that are established early in infection, consistent with distinct underlying immunological mechanisms and with potential relevance for pathogen-specific diagnostic and therapeutic strategies.

An important finding of this study was the generally weak coupling between viral load and systemic inflammatory activity. Although higher viral loads have previously been linked to increased cytokine production in dengue infection, this pattern was largely absent here. CHIKV was the exception, with IFN-α2, IL-6, IL-8 and MCP-1 all correlating positively with viral load, consistent with a closer relationship between viral replication and inflammatory mediator production in this infection than in the other three. DENV1 showed a single significant correlation, a negative association between viral load and IFN-γ, and OROV showed negative correlations with IL-12p70 and with IL-1β; DENV2 showed no significant correlation for any mediator assessed. This largely uncoupled pattern suggests that, once triggered, the inflammatory response is driven predominantly by early host innate immune mechanisms, including pattern-recognition receptor signalling and inflammasome activation, rather than by the magnitude of circulating virus.

Several limitations should be considered when interpreting these findings. The sample size was relatively modest, which limits statistical power and the ability to detect weaker associations or to perform more comprehensive multivariable analyses. Samples were also collected during the early acute phase (1–4 days after symptom onset for DENV and OROV, and up to 6 days for CHIKV), a window in which viral load falls rapidly while cytokine concentrations may persist or continue to rise; this temporal mismatch is likely to attenuate correlations between viral load and inflammatory mediators in a cross-sectional design. These observations should therefore be regarded as exploratory and warrant confirmation in larger, longitudinal studies with serial sampling across the course of infection.

Patients were not followed prospectively after sample collection, and no systematic clinical outcome data were available for the cohort. The study was not designed to evaluate the predictive value of the mediators measured, and whether these profiles anticipate progression to severe disease remains to be tested.

Overall, these findings indicate that circulating cytokines are better interpreted as markers of host immune activation than as direct surrogates of viral replication, which may contribute to differential diagnosis and to the rational design of pathogen-specific therapeutic strategies.

## Supplementary Information

Below is the link to the electronic supplementary material.

Supplementary figure 1 Supplementary Fig. 1. Cytokine and chemokine profile in Dengue, Oropouche and Chikungunya virus-infected patients stratified by days after symptom onset. Boxplots with dots of serum cytokines and chemokines concentrations (pg/mL) in healthy controls (HC) and patients with Oropouche (**A**), Chikungunya (**B**), Dengue 1 (**C**), Dengue 2 (**D**) per every 2 days after onset of symptoms, with up to 3-4 days in Oropouche and Dengue and up to 5-6 in Chikungunya infected patients. Statistical significance was determined using Wilcoxon Rank-Sum test and p-values were corrected using Benjamini-Hochberg FDR procedure (*: *p* < 0.05, **: *p* < 0.01, ***: *p* < 0.001, and ****: *p* < 0.0001) (PNG 761 kb)

Supplementary Material 1. Spreadsheets file containing detailed statistical results. (Sheet 1 – Wilcoxon) show the results from the Wilcoxon Rank-Sum cytokines differences analyses between controls, CHIKV, DENV1, DENV2 and OROV patients, with Hodges-Lehmann effect sizes and FDR correction by Benjamini Hochberg. (**Sheet 2 – Outliers**) summarises outliers obtained through the robust MAD score analysis showing which samples were removed in each cytokine comparison

Supplementary figure 2 Supplementary Fig. 2. Correlation and network correlation analysis in Oropouche, Chikungunya and Dengue 1 and Dengue 2 infected patients. Spearman’s rank correlation (**A**) and Spearman’s rank correlation difference networks (**B**) relationship plots where nodes represent cytokines/chemokines and edges represent the strength of the relationship between them expressed as the Spearman’s ρ (**A**) and Δρ (**B**). Statistical significance was corrected using Benjamini-Hochberg FDR procedure for Spearman’s rank correlations and statistical significance for the difference networks was measured by nonparametric bootstrapping with 1,000 permutations and Benjamini-Hochberg corrected afterwards. Only significant relationships (adjusted *p*-values < 0.05) are shown (PNG 854 kb)

High Resolution Image (TIFF 65.9 MB)

Supplementary figure 3 Supplementary Fig. 3. Cycle threshold (Ct) differences as an indirect measure of viral load in Oropouche, Chikungunya, Dengue 1 and Dengue 2 infected patients stratified by days. Boxplots with dots showing the difference in Ct, as determined by RT-qPCR, grouped by every two days after symptom onset in each arboviral infection. Statistical significance was determined using Wilcoxon Rank-Sum test and p-values were corrected using Benjamini-Hochberg FDR procedure (*: *p* < 0.05, **: *p* < 0.01, ***: *p* < 0.001, and ****: *p* < 0.0001) (PNG 228 kb)

High Resolution Image (TIFF 127 MB)

High Resolution Image (TIFF 11.4 MB)

## Data Availability

https://github.com/cfantecelle/stats_arbv.
